# Development and clinical evaluation of a novel SHERLOCK test for *Mycoplasma genitalium*

**DOI:** 10.1128/spectrum.00445-25

**Published:** 2025-08-20

**Authors:** Ryuha Omachi, Kazuo Imai, Akihiro Sato, Masashi Tanaka, Hitomi Mizushina, Keita Takeuchi, Takuya Maeda

**Affiliations:** 1 Department of Clinical Laboratory, Saitama Medical University Hospital 73443, Iruma, Saitama, Japan; 2 KARADA Internal Medicine Clinic, Tokyo, Japan; University of Rome, Rome, Italy

**Keywords:** SHERLOCK, CRISPR, Mycoplasma, urethritis, POCT

## Abstract

*Mycoplasma genitalium* (MG) is a sexually transmitted pathogen associated with urethritis. Nucleic acid amplification tests are the gold standard for its diagnosis but often require specialized equipment, which limits their use in point-of-care testing. This study aimed to develop a rapid, sensitive detection method for MG using a specific high-sensitivity enzymatic reporter unlocking (SHERLOCK) test, which combines isothermal recombinase polymerase amplification and a clustered regularly interspaced short palindromic repeats (CRISPR)-Cas13a reaction. We developed a novel SHERLOCK test targeting the *Mg219* gene in MG. The SHERLOCK method was evaluated using 128 first-void urine samples collected from male patients who were suspected of MG urethritis in Japan. The results of SHERLOCK were compared to those of the cobas TV/MG test and in-house quantitative PCR. SHERLOCK was optimized for use with crude DNA extracted from clinical urine samples. The results were detected via a lateral flow assay, allowing for visual interpretation within 60 min. The method demonstrated a limit of detection of 10 copies/reaction and showed no cross-reactivity with other pathogens. In clinical evaluations of 128 urine samples, SHERLOCK showed an overall agreement rate of 91.4% with the cobas TV/MG PCR test; the positive and negative agreement rates were 79.6 and 100%, respectively. SHERLOCK showed superior performance to quantitative PCR. This study demonstrates that the novel SHERLOCK assay for MG has potential as a point-of-care test in the clinical setting. Further evaluation in prospective studies is needed to confirm its clinical value.

**IMPORTANCE:**

*Mycoplasma genitalium* (MG) is a causative agent of sexually transmitted infections and is associated with urethritis and prostatitis in men. To prevent the transmission of MG, it is essential to identify infected individuals through diagnostic testing and provide appropriate treatment. Nucleic acid amplification tests are commonly used for MG diagnosis in the clinical setting, but the point-of-care testing (POCT) for MG remains limited. In this study, we developed a novel nucleic acid amplification test—specific high-sensitivity enzymatic reporter unlocking (SHERLOCK)—for MG, combining crude DNA extraction with a lateral flow assay. Our SHERLOCK assay successfully detected MG in approximately 1 h, with a detection limit of 10 copies/reaction. Clinical evaluations using urine samples showed a high agreement rate with the cobas TV/MG test. SHERLOCK is expected to be a useful tool for POCT for MG.

## INTRODUCTION

*Mycoplasma genitalium* (MG) is a causative agent of sexually transmitted infections and is associated with urethritis and prostatitis in men and cervicitis and pelvic inflammatory disease in women (1–4). Several reports indicate a global increase in its antimicrobial resistance rate (5), and, as a result, MG has garnered significant attention in recent years.

To prevent the transmission of MG, it is necessary to identify infected individuals through diagnostic testing and provide appropriate treatment. However, the diagnosis of MG remains challenging. Culture-based diagnostics for MG are not used clinically because its isolation is difficult, requiring co-cultivation with mammalian cells and taking more than 6 months to grow *in vitro* (6, 7). Moreover, the success rate of culture isolation is low (6, 7). Serological tests are difficult to develop due to the significant heterogeneity and antigenic variation among MG isolates (8, 9). Moreover, MG shares antigenic similarities with *Mycoplasma pneumoniae*, a genetically related species, which can compromise the specificity of serological tests (10). The amount of MG bacteria in clinical samples is low (8.0–50.0 × 10^4^ copies/mL in urine), making it difficult to achieve sufficient sensitivity with antigen-based tests (11). Therefore, nucleic acid amplification tests, which have high sensitivity and specificity, are used for MG diagnosis in the clinical setting (12–14).

Several nucleic acid amplification tests for MG have been developed, including the Aptima *Mycoplasma genitalium* assay (Hologic, Inc., San Diego, CA), which uses transcription-mediated amplification to target MG RNA via the Panther system (12). Another option is the cobas TV/MG test (Roche, Basel, Switzerland), which uses multiplex real-time (TaqMan) PCR for the simultaneous detection of *Trichomonas vaginalis* and MG (13–15). Among these, only the cobas TV/MG test is covered by health insurance in Japan and is clinically available in 2024. The cobas TV/MG test has very high sensitivity (85.0–100%) and specificity (96.0–99.8%) but it requires special equipment (14). As a result, it is mainly performed at testing centers, and due to sample transportation time and test scheduling intervals, it often takes several days to obtain results. An alternative molecular test that is rapid, easy to perform in the clinical setting, and suitable for point-of-care testing (POCT) is desirable for the diagnosis of MG.

Recently, a novel nucleic acid amplification test, specific high-sensitivity enzymatic reporter unlocking (SHERLOCK) (16, 17), has gained attention for POCT of infectious diseases. This method combines isothermal recombinase polymerase amplification (RPA) (18) with clustered regularly interspaced short palindromic repeats (CRISPR)–Cas13a for highly sensitive and specific nucleic acid detection. SHERLOCK operates entirely at 37°C, and the results can be interpreted visually when combined with a lateral flow assay, with a reaction time of less than 60 min (16, 17). Therefore, SHERLOCK does not require any special equipment and is expected to be a highly convenient testing method in the clinical setting. However, SHERLOCK for MG has not been reported to date.

This study aimed to develop and evaluate a simple, rapid, and sensitive detection system for MG using SHERLOCK for potential use in POCT in the clinical setting.

## MATERIALS AND METHODS

### Clinical sample collection and DNA extraction

**Fig 1:**
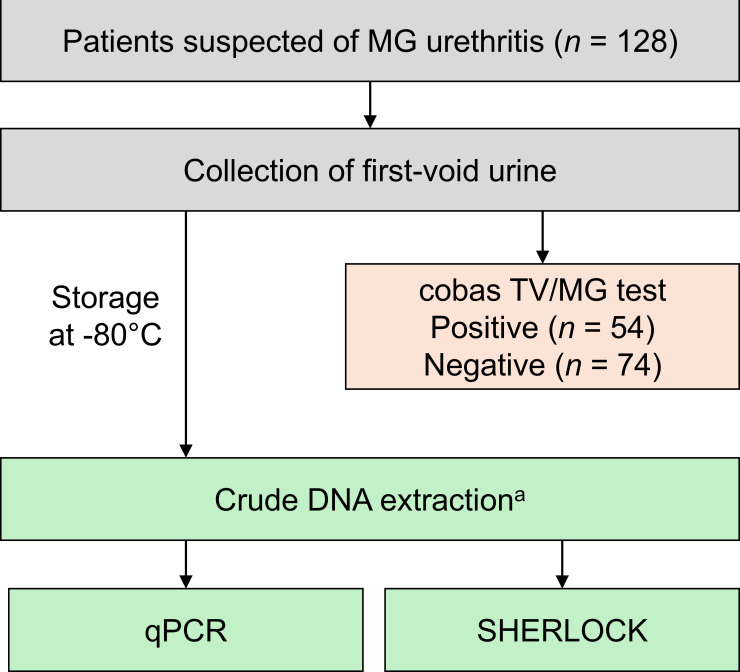
Study patients, sampling, and testing. ^a^DNA was extracted using a MightyPrep Reagent for DNA (Takara Bio, Inc.) with first-void urine samples.

First-void urine samples were collected from 128 male patients suspected of having MG urethritis who visited the KARADA Internal Medicine Clinic (Tokyo, Japan) and KARADA Internal Medicine Clinic Shibuya (Tokyo, Japan) between 2023 and 2024. First-void urine samples were divided into two portions. One portion was transferred to a cobas PCR Media Tube (Roche), and the cobas TV/MG test was subsequently performed by FML HOLDINGS, Inc. (Hiroshima, Japan). According to the results of the cobas TV/MG test, 54 patients were positive for MG, and 74 patients were negative (Fig. 1). The other portion was stored at −80°C before further use. DNA was extracted from the residual urine sample using the crude DNA extraction method with the MightyPrep Reagent for DNA (Takara Bio, Inc., Shiga, Japan). After centrifuging 1 mL of thawed urine sample at 10,000 × *g* for 10 min, the supernatant was discarded, and 50 µL lysis buffer was added. The sample was heated at 95°C for 10 min and centrifuged at 13,000 × *g* for 3 min. The supernatant was then used for testing.

### RPA primer and CRISPR RNA design

The *Mg219* gene (GenBank accession no. NC_000908.2), which is specific to MG and highly conserved across species, was selected as the target for detection. RPA primers for *Mg219* were designed using Primer3 (https://bioinfo.ut.ee/primer3-0.4.0/) according to the “Guidelines for RPA Primers and Probes” provided by TwistDx (Maidenhead, UK). Cross-primer dimers between the forward and reverse primers were analyzed using the Multiple Primer Analyzer (Thermo Fisher Scientific, Waltham, MA). The CRISPR RNA (crRNA) required for Cas13a to recognize the target sequence was designed using Cas13Design (https://cas13design.nygenome.org/). The primers and crRNA used in this study are shown in Table 1.

**TABLE 1:** Primers, probes, and crRNA used in this study^*a*^

Name	Sequence (5′–3′)	Application
*Mg219* RPA F1	ATGAAGAGTTAATCTCTGTTCCAGTTTTACTAAT	RPA, SHERLOCK
*Mg219* RPA F2	AAGAGTTAATCTCTGTTCCAGTTTTACTAATC	RPA
*Mg219* RPA F3	ATTAAAAGTAGTGACTTTATTGGTCATGAAGAGTT	RPA
*Mg219* RPA F4	GAAGAGTTAATCTCTGTTCCAGTTTTACTAAT	RPA
*Mg219* RPA R1	TTAGGTTTTGTTTGTTTAGTTGATTTACTAGTTG	RPA
*Mg219* RPA R2	TAGGTTTTGTTTGTTTAGTTGATTTACTAGTTG	RPA
T7 promoter-*Mg219* RPA R1	GAAATTAATACGACTCACTATAGGG-TTAGGTTTTGTTTGTTTAGTTGATTTACTAGTTG	SHERLOCK
RNA probe (BHQ)	FAM-UUUUU-BHQ1	SHERLOCK
RNA probe (biotin)	FAM-UUUUU-biotin	SHERLOCK
crRNA	GAUUUAGACTACCCCAAAAACGAAGGGGACUAAAACCCAGUUAAAGCAAAACCAAAAGC	SHERLOCK
crRNA template	GCTTTTGGTTTTGCTTTAACTGGGTTTTAGTCCCCTTCGTTTTTGGGGTAGTCTAAATCCCCTATAGTGAGTCGTATTAATTTC	*In vitro* transcription
T7 promoter	GAAATTAATACGACTCACTATAGGG	*In vitro* transcription
*Mg219*F	CATAGTTCATTATGCGCACCAGTTACTTG	qPCR
*Mg219*R	CTCTTTAACAACAGGGGTTGGGATTAG	qPCR
*Mg219* probe	FAM-GGTGTGGATCGAGCGGC-BHQ1	qPCR

^*a*^ Underlined text indicates the T7 promoter sequence.

### *In vitro* RNA transcription and purification of crRNA

crRNA was synthesized by *in vitro* transcription using a crRNA template DNA. The crRNA template oligonucleotide consisted of the T7 promoter sequence, the LwCas13a direct repeat sequence, and a spacer sequence complementary to the target RNA. This oligonucleotide was synthesized by Eurofins Genomics (Tokyo, Japan). The crRNA template oligonucleotide and T7 primer were annealed under the following conditions: 95°C for 2 min, followed by slow cooling to 4°C at a ramp rate of 0.08°C/s. crRNA was synthesized using a HiScribe T7 Quick High Yield RNA Synthesis Kit (New England Biolabs, Ipswich, MA). After synthesis, crRNA was purified using a miRNeasy Micro Kit (QIAGEN, Hilden, Germany). The concentration of purified RNA was measured using a Qubit RNA BR Assay Kit (Invitrogen, Carlsbad, CA), and it was stored at −80°C until use.

### qPCR for *Mg219*

qPCR for the *Mg219* gene of MG was performed as described previously, with slight modifications (19). The fluorescent dye of the probe was changed from Cy5 to FAM (Table 1). qPCR was conducted using a QuantiTect Probe PCR Kit (QIAGEN). The final reaction volume was 20 µL, with 2 µL DNA template. qPCR was carried out on a QuantStudio 5 (Applied Biosystems, Waltham, MA), with the following thermal conditions: initial activation at 95°C for 15 min, followed by 45 cycles of denaturation at 94°C for 15 s and annealing/extension at 60°C for 1 min. Ten-fold serial dilutions of AMPLIRUN MYCOPLASMA GENITALIUM DNA CONTROL (Vircell S.L., Granada, Spain), ranging from 1.0 to 10,000 copies/reaction, were used to estimate the MG DNA copy number in the specimens. The reactions were performed in duplicate.

### *Mg219* SHERLOCK

RPA for *Mg219* was performed using a TwistAmp Liquid Basic Kit (TwistDx), with a final reaction volume of 20 µL and 2 µL DNA template. The RPA reaction was conducted at 37°C for 30 min. The LwCas13a reaction had a total volume of 20 µL, comprising 2 µL of 10× Cas13 reaction buffer (SignalChem Diagnostics, Richmond, Canada), 1 µL of 10 mM NTP buffer mix (New England Biolabs), 0.5 µL of T7 polymerase mix from a HiScribe T7 Quick High Yield RNA Synthesis Kit (New England Biolabs), 0.5 µL of 40 U/µL RNase inhibitor (Takara Bio, Inc.), 1 µL of 900 nM LwCas13a (SignalChem Diagnostics), 1 µL of 2,500 nM FAM-UUUUU-BHQ RNA probe, 1 µL of 450 nM crRNA, 2 µL of RPA product, and 11 µL of RNase-free water. The reaction was conducted at 37°C.

The amplified double-stranded DNA product, generated by the RPA primers with a T7 promoter sequence, is transcribed into single-stranded RNA by T7 transcription. When the target nucleic acid is present, the LwCas13a-crRNA complex binds to the single-stranded RNA target, activating and cleaving the RNA probe. This cleavage can be detected as a fluorescent signal. For fluorescence detection, the reaction was carried out using a QuantStudio 5 (Applied Biosystems), with FAM fluorescence measured every 1 min.

Tenfold serial dilutions of AMPLIRUN MYCOPLASMA GENITALIUM DNA CONTROL (Vircell S.L.) ranging from 1.0 to 1,000 copies/reaction were used to determine the preliminary limit of detection for the SHERLOCK reactions, performed in duplicate. In addition, the cross-reactivity of SHERLOCK with DNA from bacteria, fungi, and viral pathogens was examined in duplicate (Table 2).

**TABLE 2:** Control DNA used in this study to assess the cross-reactivity of *Mg219* SHERLOCK^*a*^

No.	Type	Species	Resource	No.
1	Bacteria	*Aggregatibacter segnis*	NBRP	GTC 15056
2	Bacteria	*Burkholderia pseudomallei*	NBRP	GTC 3P0056
3	Bacteria	*Chlamydia trachomatis*	Vircell S.L.	MBC012
4	Bacteria	*Corynebacterium diphtheriae*	NBRP	GTC 00263T
5	Bacteria	*Enterobacter cloacae*	NBRP	GTC 09428
6	Bacteria	*Escherichia coli*	NBRP	GTC 00503
7	Bacteria	*Haemophilus haemolyticus*	NBRP	GTC 15009T
8	Bacteria	*Haemophilus influenzae*	NBRP	GTC 14202T
9	Bacteria	*Haemophilus parainfluenzae*	NBRP	GTC 02091
10	Bacteria	*Klebsiella pneumoniae*	NBRP	GTC 15087
11	Bacteria	*Legionella pneumoniae*	NBRP	GTC 00745
12	Bacteria	*Moraxella catarrhalis*	NBRP	GTC 01544T
13	Bacteria	*Mycoplasma hominis*	Vircell S.L.	MBC084
14	Bacteria	*Mycoplasma pneumoniae*	Clinical isolate	Not applicable
15	Bacteria	*Neisseria gonorrhoeae*	Vircell S.L.	MBC075
16	Bacteria	*Proteus mirabilis*	NBRP	GTC 14752
17	Bacteria	*Pseudomonas aeruginosa*	NBRP	GTC 13866
18	Bacteria	*Serratia marcescens*	NBRP	GTC 03891
19	Bacteria	*Staphylococcus aureus*	NBRP	GTC 01180
20	Bacteria	*Staphylococcus epidermidis*	NBRP	GTC 01871
21	Bacteria	*Stenotrophomonas maltophilia*	NBRP	GTC 00090T
22	Bacteria	*Streptococcus agalactiae*	NBRP	GTC 08356T
23	Bacteria	*Streptococcus mutans*	NBRP	GTC 01700T
24	Bacteria	*Streptococcus pneumoniae*	NBRP	GTC 00980
25	Bacteria	*Streptococcus pyogenes*	NBRP	GTC 00262T
26	Bacteria	*Treponema pallidum*	Vircell S.L.	MBC109
27	Bacteria	*Ureaplasma parvum*	Vircell S.L.	MBC133-R
28	Bacteria	*Ureaplasma urealyticum*	Vircell S.L.	MBC112
29	Fungi	*Aspergillus fumigatus*	NBRP	IFM 4942
30	Fungi	*Candida albicans*	NBRP	IFM 4949
31	Fungi	*Candida glabrata*	NBRP	IFM 5489
32	Fungi	*Candida krusei*	NBRP	IFM 5462
33	Fungi	*Candida parapsilosis*	NBRP	IFM 5464
34	Fungi	*Candida tropicalis*	NBRP	IFM 5446
35	Fungi	*Cryptococcus gatii*	NBRP	IFM 61880
36	Fungi	*Cryptococcus neoformans*	NBRP	IFM 5505
37	Fungi	*Epidermophyton floccosum*	NBRP	IFM 56842
38	Fungi	*Fonsecaea pedrosoi*	NBRP	IFM 4856
39	Fungi	*Madurella mycetomatis*	NBRP	IFM 46458
40	Fungi	*Malassezia furfur*	NBRP	IFM 40081
41	Fungi	*Microsporum ferrugineum*	NBRP	IFM 41980
42	Fungi	*Nocardia farcinica*	NBRP	IFM 0138
43	Fungi	*Spirotrichum purpureum*	NBRP	IFM 55089
44	Fungi	*Trichophyton rubrum*	NBRP	IFM 40732
45	Fungi	*Trichophyton violaceum*	NBRP	IFM 46913
46	Protozoa	*Trichomonas vaginalis*	NBRP	Kurume strain
47	Virus	*Herpes simplex virus-1*	Vircell S.L.	Not applicable
48	Virus	*Herpes simplex virus-2*	Vircell S.L.	Not applicable

^*a*^ NBPR, National BioResource Project.

### *Mg219* SHERLOCK with a lateral flow assay

**Fig 2:**
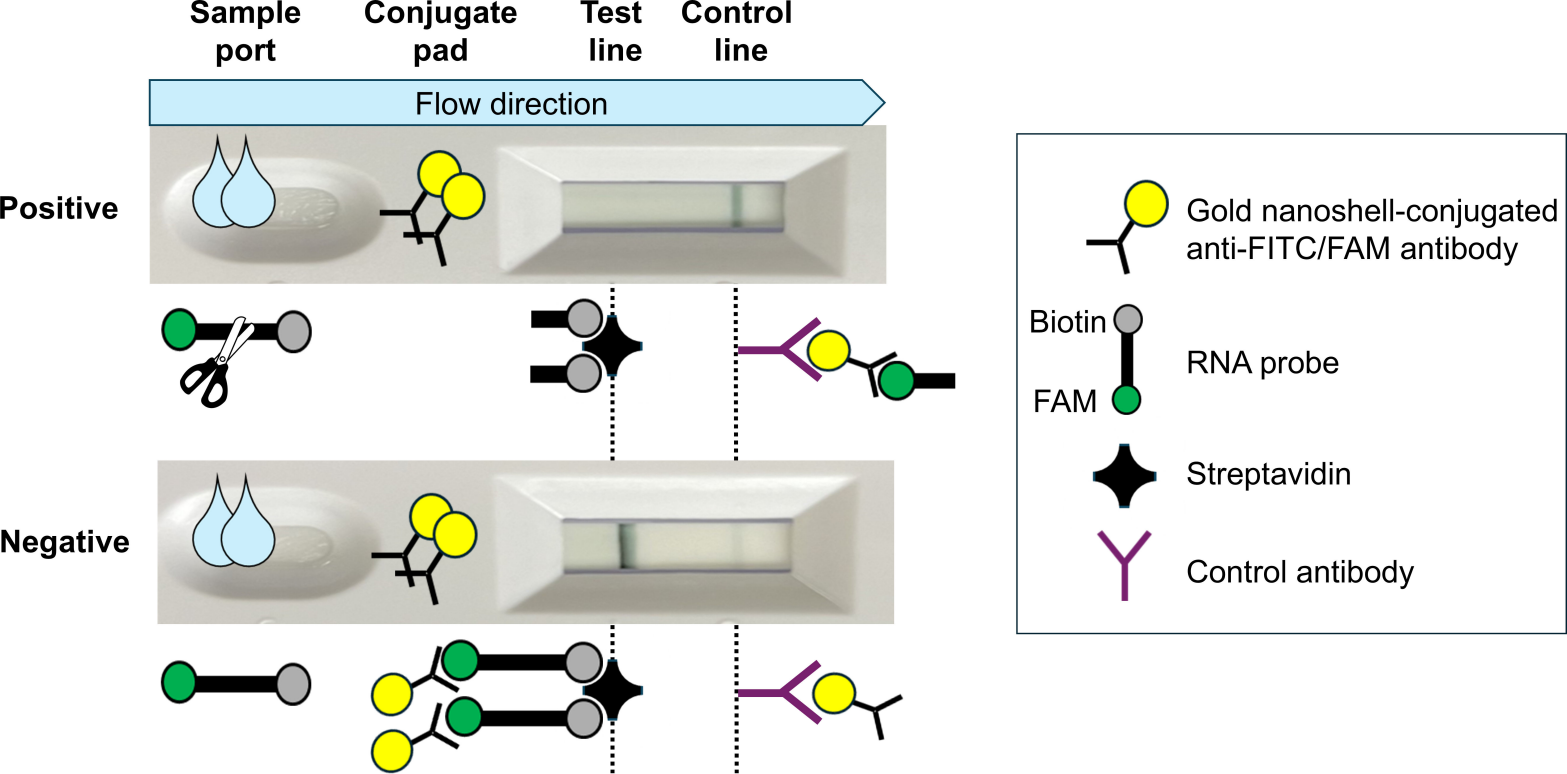
SHERLOCK with a lateral flow kit. The SHERLOCK reaction products were applied to an AmpliDetect Nucleic Acid Lateral Flow Kit (nanoComposix). In a negative SHERLOCK result, the RNA probe remains uncleaved, and a band appears at the test line. In a positive result, the probe is cleaved by LwCas13a activity, and no band appears. The control line binds residual conjugate and validates the assay.

For visual detection, the FAM-UUUUU-BHQ RNA probe in the LwCas13a reaction was replaced with the FAM-UUUUU-biotin RNA probe, and the crRNA concentration was increased from 450 to 2,000 nM. The LwCas13a reaction products were then added to an AmpliDetect Nucleic Acid Lateral Flow Kit (nanoComposix, San Diego, CA). AmpliDetect is a universal detection method for nucleic acid sequences labeled with fluorescein (FITC/FAM) and biotin. The assay is based on a lateral flow sandwich format using gold nanoshells for enhanced sensitivity. On the conjugate pad, the anti-FAM/FITC-gold nanoshell conjugate captures the FAM at the 5′-end of the RNA probe. The conjugate-sample complex then travels up the strip, where the streptavidin test line recognizes and captures the biotin. The control line, consisting of a secondary antibody, binds any residual conjugate, validating the assay. In a negative SHERLOCK result, the RNA probe remains uncleaved. When the reaction mixture is applied to the lateral flow strip, the gold nanoshells bind to the FAM side of the uncleaved RNA probe and subsequently bind to the test line, resulting in the appearance of a band at the test line. In a positive SHERLOCK result, the RNA probe is cleaved by LwCas13a, and no band appears at the test line, as the gold nanoshells can no longer bind to the cleaved probe (Fig. 2). The results are observed visually by examining the test lines with the naked eye.

### Statistical analysis

Continuous variables are expressed as the mean ± standard deviation or median with interquartile range and were compared using a *t*-test or Wilcoxon rank-sum test for parametric and non-parametric data, respectively. Categorical variables are expressed as numbers (%) and were compared using a chi-squared test or Fisher’s exact test, as appropriate. Positive, negative, and total agreement rates, along with their 95% confidence intervals, were calculated. All statistical analyses were performed using R (v 4.0.0; R Foundation for Statistical Computing, Vienna, Austria (http://www.R-project.org/)).

## RESULTS

### RPA primer selection

A combination of four forward primers and two reverse primers targeting *Mg219* (Table 1) was tested using a positive control of MG genomic DNA (1,000 copies/reaction). After the RPA reaction at 37°C for 30 min, the amplification results were analyzed via electrophoresis. The combination of forward primer F1 and reverse primer R1 resulted in the brightest band under ultraviolet light (Fig. S1). Therefore, this combination was selected for further experiments. As a result, the T7 promoter sequence was appended to the 5′-end of RPA primer R1, establishing it as the RPA primer for SHERLOCK (Table 1).

### *Mg219* SHERLOCK optimization and sensitivity and specificity measurement

**Fig 3:**
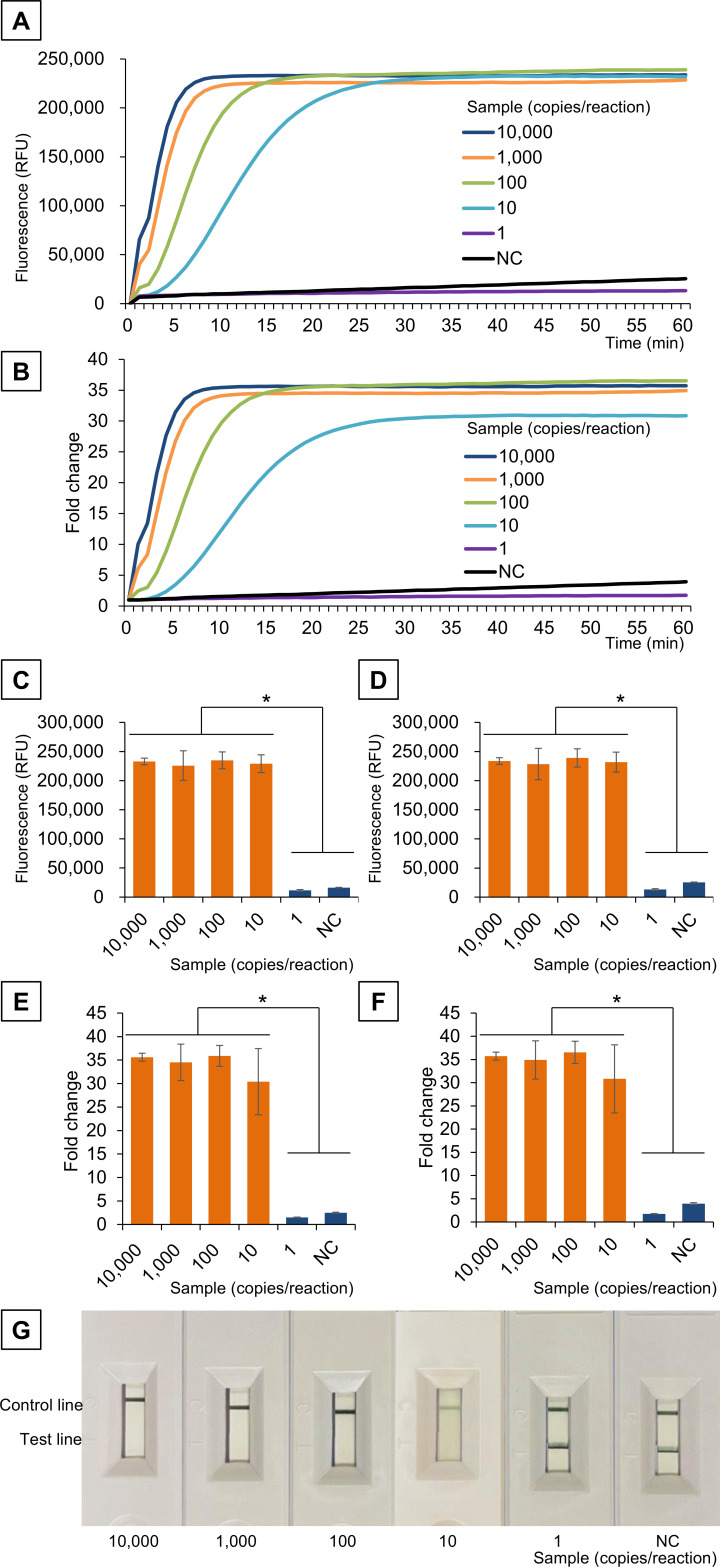
*Mg219* SHERLOCK. Fluorescent detection using a real-time PCR system (**A and B**). Fluorescence at 30 min (**C and E**) and 60 min (**D and F**). Fluorescence fold change was calculated relative to the baseline. Visual detection using a lateral flow kit (**G**). *P < 0.001. NC, no-template control; RFU, relative fluorescence units.

The LwCas13a reaction time was optimized using a positive control and no-template control. The RPA product (2 µL), generated using primers F1 and R1 (with the T7 promoter sequence added to the 5′ end), was added to the LwCas13a reaction mix, followed by FAM fluorescence detection at 37°C for 60 min. Relative fluorescence was significantly higher in the positive control with 10,000 to 10 copies/reaction compared to the positive control with one copy/reaction and the no-template control (*P* < 0.001) (Fig. 3A through F). The maximum fluorescence level was reached at 30 min after the reaction started (Fig. 3A through F). Therefore, the optimal SHERLOCK reaction time was determined to be 30 min, with a sensitivity of 10 copies/reaction. To evaluate the effect of crude DNA extraction reagents, we conducted repeated tests using 20 samples containing 10 copies/reaction—at the threshold of the limit of detection—comparing SHERLOCK with and without the addition of 2 µL crude DNA extraction reagent. SHERLOCK without the reagent yielded positive results in 15 out of 20 samples, whereas SHERLOCK with the reagent added yielded positive results in 17 out of 20 samples. A chi-squared test revealed no significant difference between the two methods (*P* = 0.48), suggesting that there was no decrease in sensitivity. No cross-reactivity was detected with microbial DNA from 48 different species, including two *Mycoplasma* species, namely, *M. hominis* and *M. pneumoniae* (Table 2).

Subsequently, the detection method was switched from fluorescence detection to visual assessment using a lateral flow assay. The disappearance of the test line was observed in the positive control with 10,000 to 10 copies/reaction (Fig. 3G), indicating that the sensitivity of SHERLOCK with the lateral flow assay remained at 10 copies/reaction, with no loss of sensitivity compared to fluorescence detection.

### Clinical performance evaluation

DNA extracted from 128 clinical urine samples (54 MG-positive and 74 MG-negative as determined by the cobas TV/MG test) was used for the clinical evaluation of *Mg219* SHERLOCK with a lateral flow assay and crude DNA. The positive agreement rate between *Mg219* SHERLOCK and the cobas TV/MG test was 79.6% (95% CI: 66.4–89.3%), the negative agreement rate was 100% (95% CI: 95.1–100.0%), and the overall agreement rate was 91.4% (95% CI: 85.1–95.6%) (Table 3). Among the 128 clinical urine samples, 39 were positive by qPCR for *Mg219*, with a median DNA copy number of 2,250 copies/mL (interquartile range: 450–9,575 copies/mL). The positive agreement rate between qPCR and cobas TV/MG was 72.2% (95% CI: 58.4–83.5%), the negative agreement rate was 100% (95% CI: 95.1–100.0%), and the overall agreement rate was 88.2% (95% CI: 81.4–93.3%) (Table 3). Discordant results between SHERLOCK and qPCR were exclusively observed in specimens that tested positive using the cobas TV/MG assay. Of the 43 specimens that were SHERLOCK-positive, 4 were found to be negative by qPCR. Conversely, 4 of the 39 qPCR-positive specimens were SHERLOCK-negative. The median qPCR copy number in the SHERLOCK-negative specimens was 0 copies/mL (interquartile range: 0–25 copies/mL), which was significantly lower than the median qPCR copy number in the SHERLOCK-positive specimens (2,250 copies/mL (interquartile range: 425–9,575 copies/mL); *P* < 0.001) (Fig. S2).

**TABLE 3:** Agreement rate between the nucleic acid amplification tests^*a*^

Test	Result	cobas TV/MG		PPA, % (95% CI)	NPA, % (95% CI)	OA, % (95% CI)
		Positive (*n* = 54)	Negative (*n* = 74)			
SHERLOCK	Positive	43	0	79.6 (66.4–89.3)	100.0 (95.1–100.0)	91.4 (85.1–95.6)
	Negative	11	74			
qPCR	Positive	39	0	72.2 (58.4–83.5)	100.0 (95.1–100.0)	88.2 (81.4–93.3)
	Negative	15	74			

^*a*^ CI, confidence interval; NPA, negative percent agreement; PPA, positive percent agreement; OA, overall agreement.

## DISCUSSION

In this study, we developed and evaluated a novel SHERLOCK-based method for the diagnosis of MG. The SHERLOCK assay successfully detected *Mg219* in approximately 1 h, with a detection limit of 10 copies/reaction. In clinical evaluations using urine samples, the agreement rate with the cobas TV/MG test was high, and it demonstrated better accuracy than qPCR targeting *Mg219*.

In this study, we selected *Mg219* as the target gene for amplification. *Mg219* has been reported as an optimal target for MG detection in qPCR-based diagnostic systems (19). Its high sequence conservation across strains facilitates the design of primers and crRNA. Furthermore, *in silico* analysis confirmed the absence of homologous sequences in other *Mycoplasma* species (19). In this study, no cross-reactivity was observed with genomic DNA from other pathogens, including *M. hominis* and *M. pneumoniae*, which are well-known human pathogens, and the negative agreement rate with the cobas TV/MG assay in clinical specimens was 100%. Although we did not assess cross-reactivity with opportunistic pathogens such as *Mycoplasma pirum, Mycoplasma fermentans, Mycoplasma penetrans,* and *Mycoplasma amphoriforme* (20–22), these organisms are extremely rare, and no *Mg219* homologs have been identified in their genomes. Therefore, the absence of testing for these species is unlikely to affect the clinical specificity of the assay.

Several nucleic acid amplification tests with potential for POCT for MG have been reported. Edwards et al. (23) developed a loop-mediated isothermal amplification assay targeting *pdhD*, with a detection limit of approximately 16 copies of MG DNA/reaction, operating at 63°C for 60 min (23). Ren et al. (2018) described an RPA assay combined with a lateral flow strip for *mgpB*, which operates at 39°C for 20 min and has a detection limit of 33.6 copies/reaction (24). These methods are comparable to the *Mg219* SHERLOCK assay developed in this study in terms of sensitivity and visual interpretation of the results. However, sensitivity evaluations in these reports used DNA purified via column-based methods (23, 24), which are time-consuming, labor-intensive, and costly, making them less suitable for POCT. In contrast, the *Mg219* SHERLOCK method successfully used crude DNA extracted with simplified procedures, maintaining high sensitivity, even with clinical urine samples. The *Mg219* SHERLOCK assay can be performed with only centrifugation, heating, and incubation steps, which are highly useful in the clinical setting.

The Aptima MG assay, which employs transcription-mediated amplification targeting *16S rRNA*, has undergone more extensive clinical evaluation than any other commercial assay. According to the manufacturer’s specifications, it has an analytical sensitivity of 0.01 colony-forming units/mL. Its clinical sensitivity and specificity are reportedly 99.13–100% and 99.57–99.96%, respectively (25, 26). The cobas TV/MG assay, which uses qPCR to detect *mgpB*, is also used widely in the clinical setting. This test demonstrates high diagnostic performance, with a sensitivity of 85.0–100% and specificity of 96.0–99.8% (14). In comparison, the positive agreement rate of the *Mg219* SHERLOCK assay developed in the present study, relative to the cobas TV/MG assay, was 79.6% (95% CI: 66.4–89.3%). While SHERLOCK offers notable advantages for POCT, it should be acknowledged that approximately 20% of positive cases may be missed. Therefore, a negative SHERLOCK result should not be used to definitively rule out infection, and follow-up with more sensitive molecular diagnostics such as Aptima MG or cobas TV/MG is recommended. Importantly, the false-negative results observed with SHERLOCK were associated with specimens that were either negative or exhibited low copy numbers by *Mg219*-targeted qPCR. This underscores the importance of proper specimen collection; specifically, the collection of first-void urine, which contains the highest bacterial load. In addition, asymptomatic individuals tend to harbor lower MG bacterial loads than symptomatic patients (27), suggesting that the SHERLOCK assay may not be suitable for use in asymptomatic populations.

The lower sensitivity of *Mg219* SHERLOCK compared to the cobas TV/MG test may be attributed to several factors: (i) differences in the target genes, as the cobas TV/MG test targets two regions of the MG genome—the *mgpB* A region (single copy) and EF region (~9 copies) (15); (ii) differences in the DNA extraction methods, as well as the fully automated process employed by the cobas TV/MG test; and (iii) variations in sample processing timing. The cobas TV/MG test uses urine samples collected immediately after voiding, while *Mg219* SHERLOCK uses samples stored at −80°C, which could cause progressive DNA degradation over time (28). Further evaluation of the diagnostic accuracy of SHERLOCK for MG in a prospective clinical study using fresh samples is warranted.

Several improvements are needed to optimize *Mg219* SHERLOCK for POCT. Simplifying the centrifugation steps in sample processing and DNA extraction, as well as reducing heating times, is important for streamlining the workflow. In addition, our method involves a two-step process—gene amplification by RPA followed by the CRISPR-Cas13a reaction—requiring multiple transfer operations and increasing the risk of aerosol contamination. A single-step SHERLOCK approach, in which both reactions are performed simultaneously, has been explored in other studies (29, 30) and could be incorporated into our system. In this study, the bands generated by the lateral flow assay were clearly visible, and no faint bands that could complicate interpretation of the results were observed. However, in clinical samples, indistinct bands may occasionally appear, and at present, the results are judged visually and subjectively. Therefore, discrepancies between evaluators may occur. The development of objective devices that utilize image analysis software to quantify band intensity for automated and standardized result interpretation may be warranted. Furthermore, this study focused on male urethritis samples, but MG also causes female cervicitis and pelvic inflammatory disease, with diagnoses typically made using vaginal and anal swabs. Future studies should assess the accuracy of SHERLOCK in diagnosing MG using other specimens.

### Conclusion

This study developed a rapid, simple detection system for MG using SHERLOCK, combining crude DNA extraction and a lateral flow assay. *Mg219* SHERLOCK is highly sensitive, specific, and capable of detecting MG DNA in urine samples, showing superior performance compared to qPCR. Further evaluations are needed, but SHERLOCK is expected to be used for POCT for MG.

## Data Availability

The data sets analyzed during the current study are available from the corresponding author on reasonable request.

## SUPPLEMENTAL MATERIAL

Supplemental material spectrum.00445-25-s0001.docx Fig. S1 and S2.

ASM does not own the copyrights to Supplemental Material that may be linked to, or accessed through, an article. The authors have granted ASM a non-exclusive, world-wide license to publish the Supplemental Material files. Please contact the corresponding author directly for reuse.
