# Supplementary material for: Development and clinical evaluation of a novel SHERLOCK test for Mycoplasma genitalium
Source: Microbiol Spectr. 2025 Aug 20;13(10):e00445-25. doi: 10.1128/spectrum.00445-25 (PMC12502767; doi:10.1128/spectrum.00445-25)
Supplement: Supplemental material — Fig. S1 and S2. [file spectrum.00445-25-s0001.docx]

**Figure S1. RPA primer pair validation by agarose gel electrophoresis.**


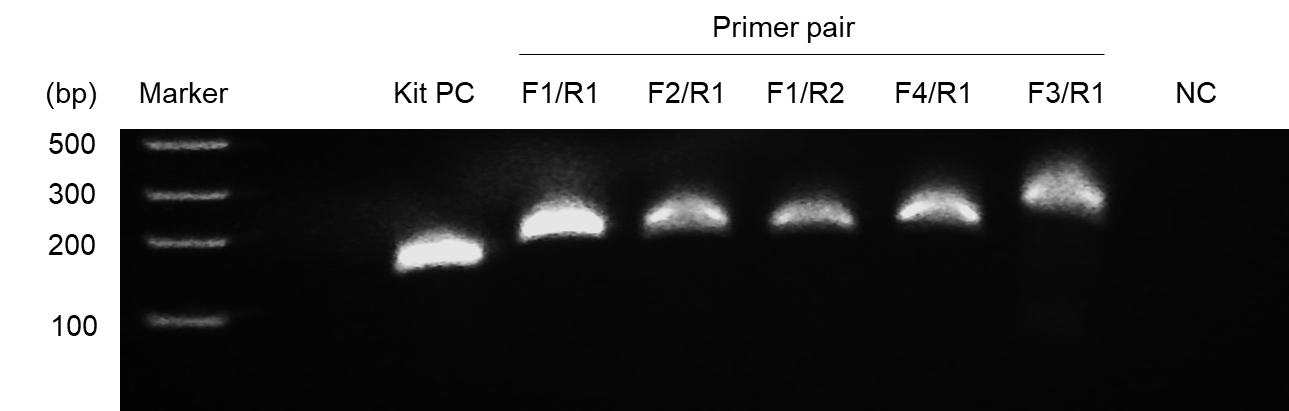


The F1/R1 primer pair produced the most intense band with 1,000 copies/μL of *Mycoplasma genitalium* genomic DNA and was selected for further analysis. Other primer combinations were excluded from consideration due to a high risk of cross-primer dimer formation. The kit positive control (PC) for the RPA reaction was provided by TwistDx. Distilled water was used as the negative control (NC).

**Figure S2. Results of SHERLOCK and qPCR in 54 cobas TV/MG-positive samples.**

**
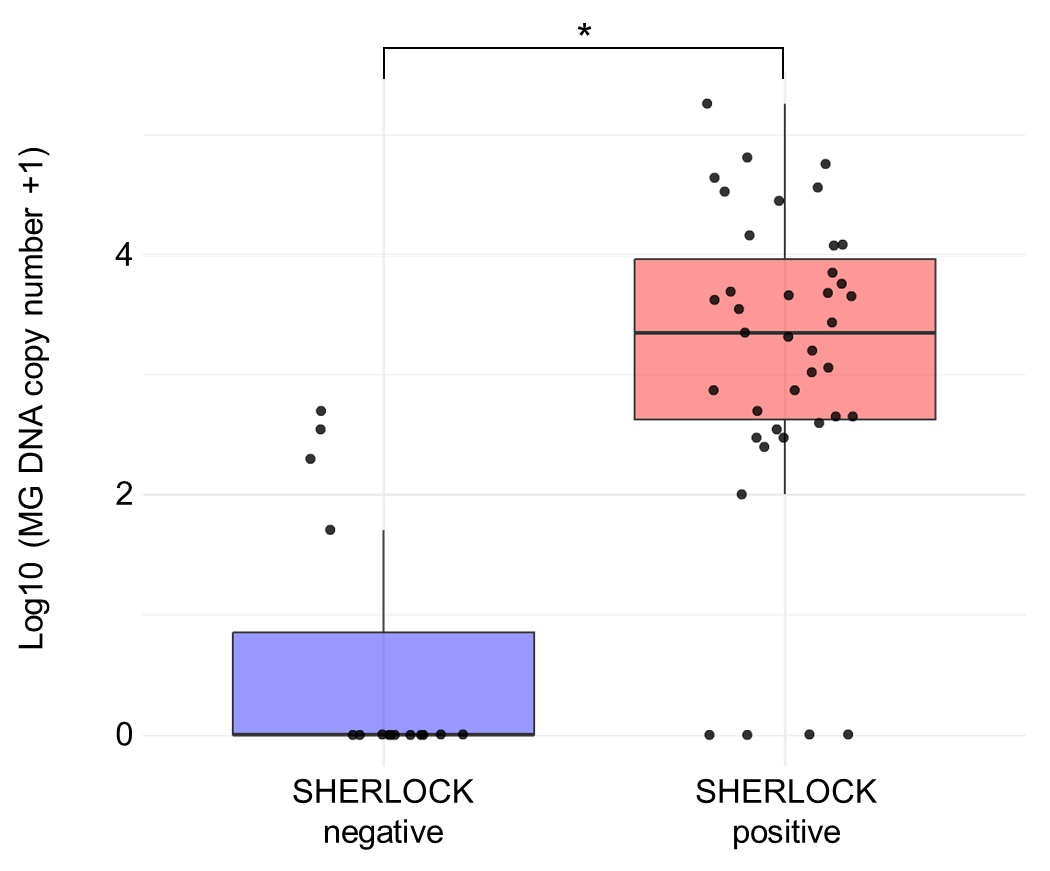
**

Negative results for the *Mg219* SHERLOCK assay (*n* = 11) were observed in clinical samples with a lower *Mycoplasma genitalium* (MG) DNA copy number than in samples with positive results (*n* = 43). Each dot shows MG DNA copy number in each sample. **p* < 0.001, Mann–Whitney U test.
